# The Design of Personalized Education Resource Recommendation System under Big Data

**DOI:** 10.1155/2022/1359730

**Published:** 2022-06-28

**Authors:** Rong Fu, Mijuan Tian, Qianjun Tang

**Affiliations:** School of Educational Sciences, Leshan Normal University, Leshan, Sichuan 614000, China

## Abstract

With the advent of the Internet and the era of big data, education is increasingly dependent on data resources to support product and business innovation, and the lack of data resources has severely limited the areas involved. As a general information filtering method, personalized recommendation systems analyze the historical interaction data between users and items to build user interest models in an environment of “information overload”, allowing users to discover and recommend information that interests them. However, the explosive growth of information in the network makes users wander in the sea of information, and it is increasingly difficult to find the information they really need, i.e., information overload. This has given rise to personalized recommendation systems, which currently have more mature applications in industries such as e-commerce, music services, and movie services. To this end, this paper studies and implements a customized educational resource recommendation system that can handle big data. The results show that the values of different similarity calculations all fluctuate with the gradual increase of the number of nearest neighbors, and the algorithm in this paper is maximum at the number of neighbors around 60; then, it is inferred that applying the calculation method to the recommendation algorithm will improve the recommendation accuracy. Therefore, education uses the concept of big data to process the huge amount of education data and find some correlations and laws in education, so as to realize “teaching according to the material, teaching according to the material”.

## 1. Introduction

With the continuous progress of China's education reform, the education informatization has made positive progress, especially since the introduction of education planning programs, the importance of education informatization has been widely recognized by the whole society [1]. The development and maturity of educational big data, especially learning analytics and data mining technologies, have provided a scientific basis for carrying out personalized and accurate learning support services [2]. In particular, the current boom in personalized education and quality education has made people more eager to access educational resources at a specific individual level [3]. It is hoped that learning contents and learning orientations suitable for the development of different learners can be provided accordingly, thus promoting their individual development and thus realizing personalized education in the true sense [4]. Recommendation systems have been successfully used in many applications and recommendation algorithms have achieved great success [5]. The amount of data entered in public curriculum resources and social discourse resources in universities will grow at a faster rate. How to maximize the academic value of so many electronic educational resources has become a critical issue that needs to be addressed [6].

The continuous development of education informatization has led to a dramatic increase in the volume of educational resources, and users need to spend a lot of time searching for educational resources in this huge amount of educational data, which leads to the phenomenon of “information overload” [7]. Digitization transforms complex and constantly changing educational information into digital or measurable data [8]. Multimedia provides more ways to exchange information in the teaching and learning process [9]. Compared to traditional data, such big data (complete data) has a huge volume of distributed unstructured data, and data analysis is shifted from the expert level to the user level [10]. Search engines, as a new method of information filtering, filter out a large amount of irrelevant information by keywords and present the query result information to the user [11]. The strategic significance of big data does not lie in the mastery of big data information, but in how to process this data professionally and efficiently through algorithms [12]. As a result, big data alliances have been formed to support big data organization management and value discovery, driven by big data.

Recommender systems are a new way of discovering information. Historical user behavior information is analyzed, user interests are modeled, and user education is recommended based on user preferences [13]. In this process, the user does not need to provide any information about the needs and the recommendation system actively sends information to the user [14]. Big data in education focuses more on the micro and individual level, which requires information collection at all times, comprehensive and objective recording of information, and the use of a large number of visualizations to help information collectors obtain accurate material [15]. The use of digital educational resource platforms provides students with high-quality and rich educational resources, creating more learning opportunities and a better learning environment, in addition to overcoming the time and space limitations of traditional education methods and providing flexible learning methods. In the recommendation system, considering the scale of user data on the Internet and the overall process of machine service, the distributed computing power of Hadoop can be used to find the optimal combination of algorithms to analyze the characteristics of users based on traditional machines.

The innovations of this paper are as follows:

1. According to the characteristics of a big data alliance, a recommendation system is designed to identify target users, process alliance data according to user needs, and recommend data resources for different types of users. The learning platform established by ontology records a large number of student data, which are analyzed by data mining tools. These data produce some rules and have a positive impact on teaching.
2. Make use of the characteristics of ontology clarity, objectivity, and scalability to construct the ontology of educational resources, as well as the ontology of student users.
3. According to the characteristics of a big data alliance, a recommendation system is designed to identify target users, process alliance data according to user needs, and recommend data resources for different types of users.

The research framework of this paper consists of five parts, and the specific arrangements are as follows.

The first part of this paper introduces the research background and significance and then introduces the main work of this paper. The second part introduces the work related to personalized educational resource recommendation systems and personalized recommendations under big data. The third part combs the recommendation methods based on similarity and the data storage methods of the recommendation algorithm so that readers of this paper can have a more comprehensive understanding of the main methods of constructing a personalized educational resource recommendation system. The fourth part is the core of the paper, which describes the analysis of personalized recommendation algorithms based on big data from two aspects: collaborative filtering recommendation algorithm analysis and personalized recommendation system test analysis. The last part of the paper is a summary of the whole work.

## 2. Related Work

### 2.1. Personalized Educational Resource Recommendation System

Social informatization and educational informatization are becoming more and more mature, social networks are gradually rising, sensor devices and mobile terminals are more and more connected to the network, and various statistical data, transaction data, interactive data, and sensor data are generated and exist. The era we live in seems to be an era of information explosion, but the emergence of massive information has overwhelmed the Internet people. There is more and more useless information, and it is difficult for users to really pay attention to themselves. A large amount of useful information is covered up and cannot be obtained by the target audience. Therefore, personalized education resource recommendation is expected to provide users with higher precision and faster recommendation services.

Gan and Zhang [16] proposed properly processing these complex and diverse structured, semistructured, and unstructured educational data to form an integrated solution covering business, technology, and IT infrastructure to process, store, manage, and analyze educational big data [16]. Zheng [17] constructs a model based on the user's past behavioral history (previous purchases, choices, item ratings) and other users' decisions and then uses the model to predict or evaluate items and then recommend a high recommendation value to the user [17]. Saito and Watanobe [18] present educational application areas and cases of big data in the United States and the challenges they face with big data application to self-learning systems as an example of educational applications of big data [18]. Ying and Boqin [19] argued that recommendation systems based on content filtering algorithms typically use a set of individual characteristics of items to calculate similar items [19]. Wu [20] argued that analyzing recorded data and discovering potential rules based on big data techniques can guide teacher education and personalized learning for teachers and students [20].

Therefore, with the increase in educational resources, building a personalized recommendation system for educational resources is a necessary link to promote students' online learning mode. How to track students' learning progress and recommend truly useful educational resources to students is a real need.

### 2.2. Personalized Recommendation under Big Data

A good recommendation algorithm can improve efficiency and serve the users, while a poor recommendation algorithm will produce various internal conflicts and the recommended information will not satisfy the users. The core of a personalized recommendation system is the recommendation algorithm, and among many recommendation algorithms, the collaborative filtering recommendation algorithm is the most researched and widely used recommendation algorithm.

Pardos et al. [21] proposed a matrix filling technique to fill the missing data with default values [21]. It is the process of processing a data source, transforming it into knowledge, and recommending it to the user. The effective fusion of different recommendation algorithms and recommendations is called the matrix filling technique. Zhu [22] uses an information-theoretic approach to measure the relevance between items and features and proposes a feature-weighted selection method to improve collaborative filtering algorithms to improve the accuracy and efficiency of recommendations [22]. Cui [23] proposed a method combining information games and Pearson correlation coefficients to improve the similarity accuracy of sparse data [23]. Singhal et al. [24] proposed using the singular value decomposition technique to reduce the dimensionality of feature vectors, reduce the time required to find nearest neighbors, and improve scaling performance based on item recommendations [24]. Sun et al. [25] proposed a method to add confidence values to the similarity calculation to improve accuracy [25].

According to the collaborative filtering algorithm, the personalized recommendation can predict which resources users may be interested in according to the historical data (exploration, scoring, etc.) and the user's community, so as to find the user's needs, increase user stickiness and loyalty, and improve user experience in a large amount of redundant information.

## 3. Main Methods of Constructing Personalized Educational Resources' Recommendation System

### 3.1. Similarity-Based Recommendation Method

**Figure 1:**
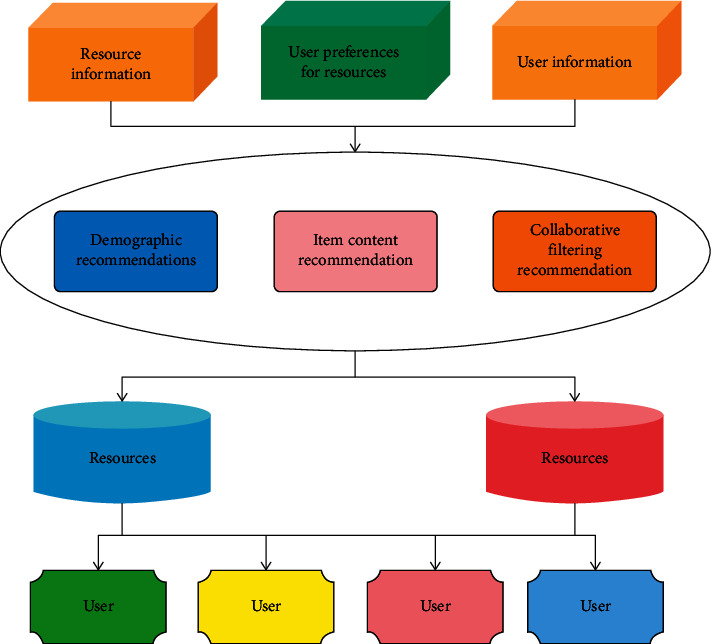
Architecture diagram of common recommendation system.

A similarity-based method is the most representative and successful recommendation method in a recommendation system [26]. The main idea is that if some educational resources or users have the same behavior in the past, they will have similar behavior in the future, so as to predict the behavior that will occur according to the behavior that has occurred; that is, educational resources or users are implicitly cooperating with each other. The nontraditional search engine will make use of the data similarity-based indexing technology in the traditional search engine. Based on the recommendation algorithm, Figure 1 shows an example of the system architecture of a common personalized recommendation system.

Firstly, the algorithm based on user similarity collects the evaluation data of other users, especially those similar to the target user, and then predicts the preference characteristics of the target user. According to the definition and theorem of attribute dependency, the dependency of class attributes relative to conditional attributes in the dataset is derived, so as to delete irrelevant attributes according to the calculation of attribute dependency [27]. Specific calculation formula of attribute dependency is as follows:

(1) $$\begin{array}{c} \gamma \left(C|A\right)=\frac{1}{\left|T\right|\left(m-1\right)}\sum_{i=1}^{n}\sum_{j=1}^{m}\frac{Tc_{i}A_{j}}{T}, \end{array}$$

where *γ*(*C*|*A*) is the attribute of *A* class dependence is *CC* and |*Tc*_*i*_*A*_*j*_| is the number of samples.

**Figure 2:**
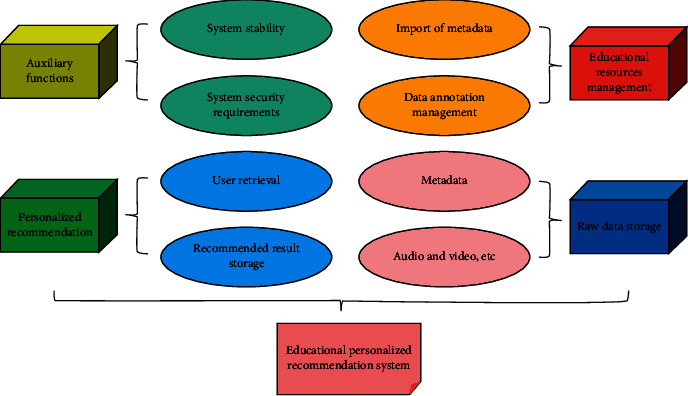
Personalized recommendation system in the process of education.

By analyzing the user data of the alliance data resource trading platform, target users are identified as those who already use the platform, and identification methods are designed for potential users outside the alliance to prepare for the introduction of the alliance platform. The functional requirements of the personalized recommendation system for the education process are shown in Figure 2.

When big data is segmented and transported to different machines for processing (each Map corresponds to a machine node), its execution efficiency must be higher than that of a single machine, and the larger the data, the higher the efficiency ratio. HDFS stores super large files by taking data blocks as the basic unit. Each file can be divided into many blocks and stored on different disks, respectively, so that a single file can be larger than the capacity of any disk in the cluster and easy for system management [28]. Assuming that *A* is a discrete attribute with *s* different values, the calculation formula of the division information amount of the information gain obtained by dividing the sample set with *A* is as follows:

(2) $$\begin{array}{c} \text{split}\left(\text{T}\right)=\sum \frac{\left|T_{i}\right|}{T}\times \;\log_{2}\left\{P\frac{\left|T_{i}\right|}{T}\right\}. \end{array}$$

Secondly, modern information extraction technology allows us to automatically extract the content or metadata information of educational resources, so we can compare the content of a given educational resource to calculate the similarity between the two resources. To eliminate the influence caused by different dimensions, this method is normalized as follows:

(3) $$\begin{array}{c} {\text{x}}^{\prime }=\frac{x-\min\left(A\right)}{\max\left(A\right)-\min\left(A\right)}. \end{array}$$

For explicit collection, through the filling in of students' basic personal information upon enrollment and the usual interactive survey of students, students are allowed to make in-depth heuristic inquiries about the knowledge they are interested in. For unified codification of shared and transactional data of alliance members, the data related to the alliance is effectively stored and managed through data processing, integration, transformation, and storage. After finding the nearest neighbors with the greatest similarity to the project, the target project is scored using the following reduction formula.

(4) $$\begin{array}{c} P\left(u,i\right)=\frac{\sum_{j\in \text{KNN}\left(i\right)}r_{ij}}{\left|\text{KNN}\left(i\right)\right|}, \end{array}$$

where |KNN(*i*)| is the number of items in the nearest neighbor set KNN(*i*) of *i*.

Then, in the collaborative filtering algorithm based on items, the similarity between items is expressed as follows:

(5) $$\begin{array}{c} \text{sim}\left(i,j\right)=\cos\left(i,j\right)=\frac{\bar{i}\cdot \bar{j}}{\bar{\left|i\right|\left|\bar{j}\right|}}, \end{array}$$

by collecting information about users and resources, processing this information using data mining-related techniques, so as to discover and analyze the intrinsic relationship between users and resources, and finally, using some recommendation strategy to select the most suitable resources for users to recommend.

Finally, in the recommendation system, the elements and dimensions of the system feature vector are first defined in advance by experts, and then all users or educational resources in the system are represented using the feature vector. In this way, the similarity between two users or educational resources can be obtained by calculating their corresponding feature vectors. The system supports dynamic changes in database size or data volumes, such as regular maintenance of resources by administrators, absorption of high-quality resources, and deletion of low-quality resources. It also adopts modular design and layered architecture design, so that the system can be applied under different operating environments and customer needs. For potential target users outside the alliance, the data resource recommendation method is designed by RSS technology, and for target users already using the platform, the dichotomous network personalized recommendation method is designed, and the recommendation evaluation method is also designed. The recommendation evaluation is mainly used to evaluate the recommendation effect of the recommendation system and provide relevant feedback to facilitate the improvement of the recommendation algorithm.

### 3.2. Recommended Algorithm Data Storage Method

The traditional social recommendation algorithm only considers the impact of the social network relationship between users on the system from the perspective of users and assumes that the items in the system are independent [29]. Its services do not only focus on keyword search but also on how to analyze the massive search records generated every day and the search history of different users. The similarity between items *i*, *j* is the comprehensive similarity of their attribute characteristics, which can be expressed as

(6) $$\begin{array}{c} \text{asim}\left(i,j\right)=\sum_{k=1}^{t}\frac{r_{k}}{1+d\left(a_{ik}+a_{jk}\right)}=\sum_{k=1}^{t}\frac{r_{k}}{1+\left|a_{ik}-a_{jk}\right|}, \end{array}$$

where *r*_*k*_/1+*d*(*a*_*ik*_, *a*_*jk*_) is the similarity of items *i*, *j* in the attribute *k*.

**Figure 3:**
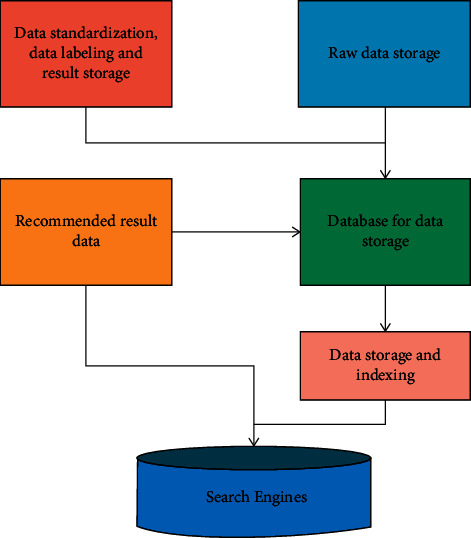
Data storage and indexing module.

However, information about the interrelationships between items in the system is also important because people will consider similar items or alternative items in some cases when choosing items. Traditional recommendation systems require explicit social network relationships between users. The data source can be imported directly into Elastic Search or Hbase, or one of the importing parties can subsequently perform data synchronization operations. The data storage and indexing module is shown in Figure 3.

The first is based on the content recommendation module; this module is relatively simple and computationally small, so the output data can be calculated in real time without saving. After the user submits the job with the command Hadoop jar, the JobClient instance uploads the job configuration information, jar file, environment variables, slice, and other related information to the distributed file system HDFS, and then JobHent notifies JobTracker through the RPC framework. The similarity between users *u*, *v* is the similarity of their combined features, which can be defined as follows:

(7) $$\begin{array}{c} \text{csim}\left(u,v\right)=\sum_{k=1}^{\text{s}}\frac{l_{k}}{1+d\left(c_{uk},c_{vk}\right)}=\sum_{k=1}^{s}\frac{l_{k}}{1+\left|c_{uk}-c_{vk}\right|}, \end{array}$$

where *l*_*k*_/1+*d*(*c*_*uk*_, *c*_*vk*_) is the similarity of *u*, *v* users on the *k* feature.

The weight of the project *i* to the user *u* is defined as follows:

(8) $$\begin{array}{c} WT\left(u,i\right)=\left(1-\alpha \right)+\alpha \frac{D_{ui}}{L_{u}}, \end{array}$$

where *D*_*ui*_*D*_*ui*_ is the time interval between visiting items *i* and *u* ‘s earliest visit to an item and *L*_*u*_ is the time span of using recommended system.

To ensure high availability and data fault tolerance in the Data Node, each block can be replicated to other server disks, with a default of 5, thus ensuring no data loss in case of the block, disk, or machine failure. Load the session logs from it to the data warehouse for offline analysis using big data analytics tools such as Hive and Pig.

Next is the user-based collaborative filtering recommendation module, which will calculate the user's *K* neighborhood and the degree of recommendation of teaching resources to the user based on the user's preference matrix, which will be calculated offline because of the large amount of calculation. The job scheduling module will initialize the job at the right time, that is, create a JobProgress object for the job to track the running status of the job, and JobProgress will create a TaskInProgress object for each Task to track the running status of each Task. This can reduce the probability of system downtime to a certain extent and further improve the stability and data tolerance of the system to cope with the increasing number of course teaching resources in the future. We back up system metadata files, while persisting file system metadata to local disk files and writing to a remotely mounted network file system. Generally, ETL (Extract, Transform, Load) operations are required on the data, even using data mining-related techniques. By introducing a temporal weight function in the prediction scoring process, the new prediction equation is

(9) $$\begin{array}{c} P_{uj}=\bar{R_{u}}+\frac{\sum {}_{v\in L}\left(R_{vj}-\bar{R_{v}}\right)\text{sim}\left(u,v\right)\cdot WT\left(u,i\right)}{\sum {}_{v\in L}\left(\left|\text{sim}\left(u,v\right)WT\left(u,i\right)\right|\right)}, \end{array}$$

where $\bar{R_{u}}$$\bar{R_{u}}$ is the average score, *R*_*vj*_ is the scoring of item *j*, sim(*u*, *v*) is the similarity between users, and *L* is the nearest neighbor set.

When only one neighbor user scores an item, it is only related to this user's score, as shown in the following formula:

(10) $$\begin{array}{c} P_{ui}=\bar{R_{u}}+R_{vi}-R_{v}. \end{array}$$

The last is based on the neural network recommendation module; when the user scores a resource, it will call the wood module on the home page recommendation neural network training, and the input feature value of the neural network is directly taken out from the database to get. The number of clicks students make using the education platform, the time they access the resources, the type of resources they access, exam records, and the time they spend doing questions are analyzed to train a student user model [30], for nonrelational massive data, such as user page stay practice, the number of clicks, learning behavior, and other massive data, by using Hadoop system in the HDFS (Distributed File Storage System) to the massive education big data storage operations. Once there are free resources, JobTracker will select a suitable task to use these resources according to a certain policy, which is done by the task scheduler. Teachers can carry out off-site synchronous interactive teaching through the big data platform and can also monitor each student's learning process and dynamically adjust the teaching content and pace according to the characteristics of students' learning behavior, tailoring high-quality personalized teaching.

## 4. Analysis of Personalized Recommendation Algorithm Based on Big Data

### 4.1. Analysis of Personalized Collaborative Filtering Recommendation Algorithm

**Figure 4:**
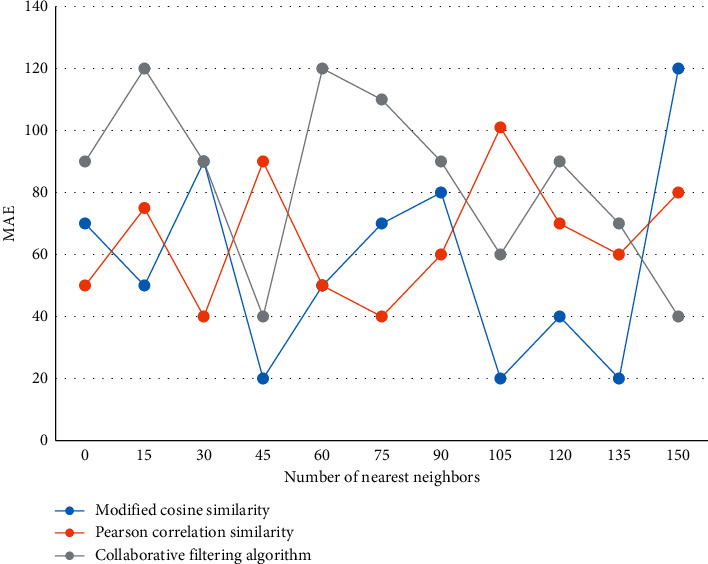
Comparison of similarity calculation methods in traditional recommendation algorithms.

The number of registered users on the Web is constantly increasing, and in the face of the complex structure of websites and the increasing number of large-scale users, recommendation algorithms will face great challenges in calculating the similarity between objects and finding the nearest neighbors of the target objects, and clustering can exactly remedy this problem. Three traditional collaborative filtering similarity measures, namely, cosine similarity, Pearson correlation similarity, and modified cosine similarity, are applied to the same platform for experiments, and the best similarity measure is selected based on the trend and applied to the collaborative filtering recommendation algorithm proposed in this paper to find the nearest neighbors of the target users. The results are shown in Figure 4.

The values of the three similarity calculations fluctuate with the gradual increase of the number of nearest neighbors. The algorithm in this paper is the largest when the number of neighbors is about 60, while the value of the Pearson correlation similarity has always been the smallest. It is inferred that applying this calculation method to the recommendation algorithm will improve the recommendation accuracy.

**Figure 5:**
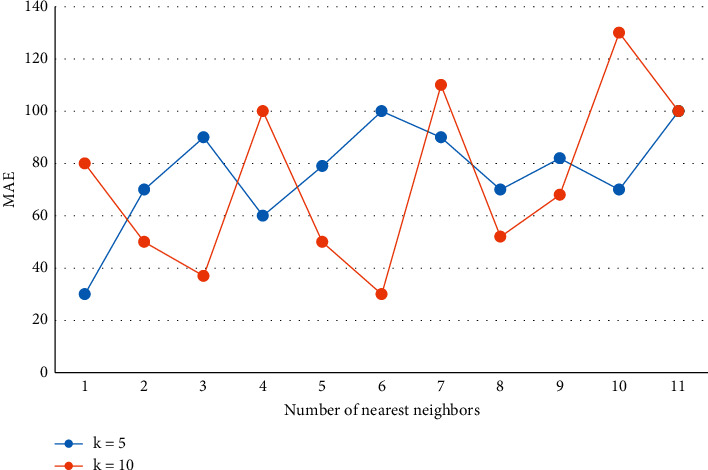
Comparison of recommendation accuracy with different cluster numbers.

The working mechanism of the collaborative filtering recommendation algorithm is to calculate user similarity based on user ratings of items, find nearest neighbors, and make recommendations to target users based on the nearest neighbors' ratings of the target items. That is, some features are extracted for each recommended object to represent this object, then the feature data of a user's past favorite (dislike) objects are used to learn this user's preferences, and finally, a set of objects with the greatest relevance is recommended for this user by comparing the user preferences obtained in the previous step with the features of the candidate objects. In order to verify the accuracy of for, *k* = 5, 10 is selected to compare the real face, while the number of neighbors is taken as the number of intervals between to, and the results are shown in Figure 5.

**Figure 6:**
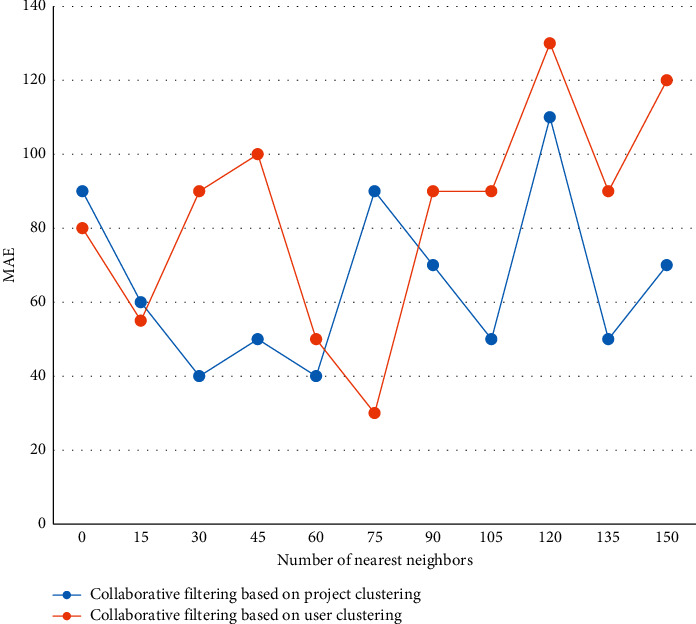
Comparison of recommendation quality of different recommendation algorithms.

First, data mining and preprocessing methods are used to preprocess and model the data that can reflect users' purchasing habits or ratings so that they can be processed by recommendation algorithms to come up with quick conclusions. In the massive resource system, searching for resources is a more convenient way to obtain resources. As the number of users using the learning resource recommendation system increases, new users are gradually transformed into regular users. By analyzing users' search keywords, we can get some users' interest points. By the iterative method, the value of each clustering center is updated one by one until the best clustering result is obtained. Discovering frequent item sets based on support degree, we first scan the transactions to discover the number of occurrences of each item and build a candidate set. Then, the support degree is assumed in advance and all frequent item sets are calculated by an iterative approach. The optimal number of clusters is taken so that the number of nearest neighbors ranges from 0 to 150 with progressively increasing intervals, and the experimental results are shown in Figure 6.

Secondly, by calculating the distance between the target and the cluster centers of several clusters that have been determined, the cluster with the closest spatial distance, that is, the most similar cluster, is selected. The association rules are analyzed from the subset of frequent item sets through the preassumed confidence. Users are divided into different clusters according to their personal portrait information (gender, age, occupation, etc.). Users in the same cluster have high similarity (specifically, they may have the same or similar interest preferences). In each cluster, an “opinion leader” is generated by calculating the average evaluation of users on educational resources to represent the preferences of users for all items in the whole cluster. If the user is interested in what subject content, these subject words are used as the user's interest model.

Finally, based on the rating data information of *K* nearest neighbor users of the target user, the items not rated by the target user are predicted to be rated and the final recommendation results are generated. After the user's learning behavior database is gradually built up, the recommendation system will start the recommendation methods of collaborative filtering and association rules and finally realize the hybrid recommendation of three recommendation methods in parallel. If the buffer is written to a certain threshold value, it will start to partition into files, during which the data will be partitioned by the number of Reduces and sorted within the partition by the same key value and then written to the output partition file. In particular, the clustering center needs to be continuously adjusted to a suitable state through computation, and then other data objects are effectively classified, which involves a large amount of distance-based computation between data volumes in this process, thus making the clustering process take a lot of time and eventually affecting the rate of clustering.

### 4.2. Test and Analysis of Personalized Recommendation System

**Figure 7:**
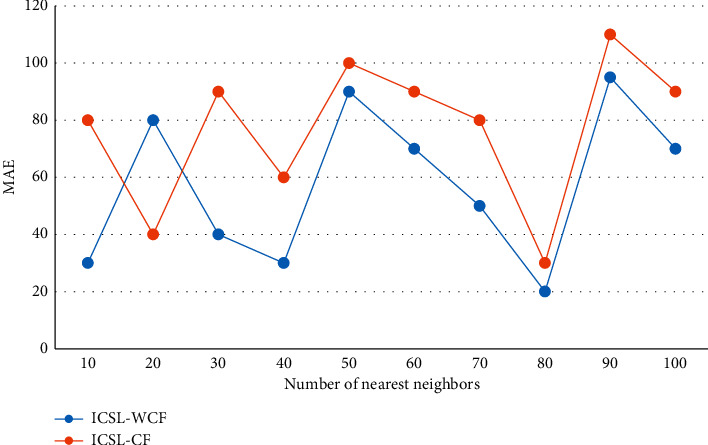
Comparison of algorithms.

The recommendation process of the detailed page of teaching resources is somewhat similar to the search process of search engines, in that it calculates the result list that is closest to the user input and close to the user preference according to the user's input and extensive habits. In this dataset, the performance difference between social recommendation systems using explicit social network relationships and implicit social recommendation systems using implicit social network relationships is compared. However, the same user's rating on a project can also use this global difference to determine its relationship with the project, manage the server's hardware resource pool through the client, and then virtualize all the hardware resources in the resource pool to generate several independent virtual machines, so as to reduce the running burden of the system. By weighting the similarity of users in each cluster, this improved algorithm can be called the ICSL-WCF algorithm, and the comparison result with ICSL-CF is shown in Figure 7.

First, the top rankings are provided to the using users and switched to personalized recommendations after waiting until a certain amount of user data is collected. A penalty factor will be added to improve the user-based collaborative filtering algorithm together with the K-means clustering algorithm, and the evaluation results are shown in Tables 1 and 2.

**Figure 8:**
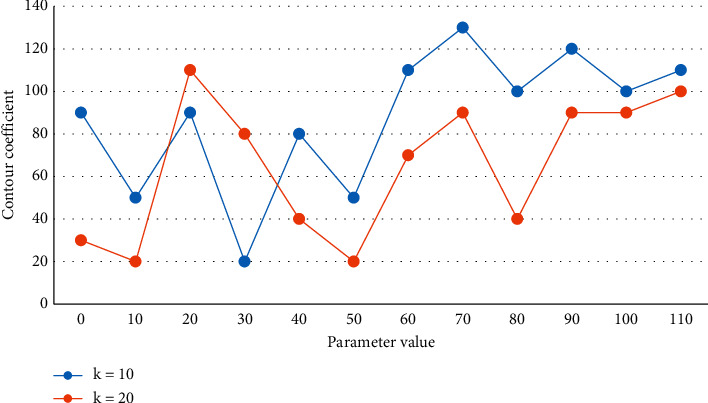
Comparison of contour coefficients of different cluster numbers.

The data is read into the Map function in parallel according to the chunking of the file in HDFS, and the Map extracts the (key, value) key-value pairs according to the actual requirements, sorts them, and submits them to the Reduce function. For image objects, we need to extract features such as color, texture, shape, and spatial relationship, while audio and video objects are generally extracted by deep learning methods for sound spectrum and video frames. This data is then used as input for the Reduce process, and the result is output to HDFS. Based on the relative preference matrix of each user, the clustering center is found. The number of clusters is selected as 10 and 20, and the contour coefficients are shown in Figure 8.

Secondly, for the information on different educational resources, it makes full use of the current resources such as teachers and experts in the school to label the resources in a corresponding way. As long as the user has a rating and can calculate its recommendation items, even if there are no brand new users, we can use the form of taking the closest user or popular average to supplement its rating and thus Slope open recommendation. This process can take full advantage of the distribution of computing resources of each node and parallel MapReduce processing, but this framework is a major problem that in the Reduce process requires all MapReduce written partition files through the network copy to the Reduce node and then the next step.

Finally, the prediction accuracy of the recommendation system implemented in this paper is statistically calculated by means of offline calculations. The method is based on the assumption that users are always interested in the most recently accessed information; i.e., data within the user's most recent fixed time frame is always analyzed and modeled, ignoring all data prior to that time window. The validation process verifies the validity of the selected subset of features on a test dataset. In the data model of the recommender system, the data generated by the user during the use of the learning resources by the learner will be stored by creating and using the learning resources database. In different application domains, the focus of people on the attribute features is different, so the size of the contribution made by different attribute features in the final recommendation varies, and this weight is generally determined by the characteristics of the relevant domain or through expert opinion.

## 5. Conclusions

With the rapid growth of today's data volume, recommendation systems have become one of the most effective ways to solve the problem of “information overload”. In the personalized recommendation system of educational resources, the user interest model is the foundation and core, and the research on the user interest model is increasing. With the rapid growth of Internet users and online information, the importance of recommendation systems is further strengthened and people's expectation of the recommendation result of recommendation system is further increased, so the recommendation accuracy of recommendation system must be improved continuously. In terms of students' self-learning, the web tracks the whole learning process of students to understand their learning methods and habits, personalities, and interests and to obtain relevant implicit data. However, data storage and nonstandardized descriptions increase the difficulty of data mining. This paper mainly designs and implements a personalized recommendation system for teaching resources, which can effectively solve the problems of a large amount of resource information, inaccurate resource allocation, scattered and difficult to share resources, duplicated resource organization, insufficient intelligence, and difficulties. It is a subsystem of the advanced computer network course faculty support system, and its main function is to provide a customized recommendation and faculty resource sharing platform. The personalized recommendation system based on big data meets functional and nonfunctional system design requirements, effectively helps solve the information overload problem, and recommends personalized educational resources in a timely manner. It effectively provides customized educational resources and supports and facilitates the promotion of education in China.

## Figures and Tables

**Table 1:** Comparison of accuracy before and after improvement.

User level	*S*1	*S*2	*S*3	*S*4
*K*-means accuracy	0.54	0.63	0.48	0.52
Comprehensive improvement accuracy	0.37	0.47	0.39	0.28
Relative improvement	0.48	0.51	0.38	0.47

**Table 2:** Comparison of recall rate before and after improvement.

User level	*S*1	*S*2	*S*3	*S*4
*K*-means accuracy	0.22	0.37	0.28	0.19
Comprehensive improvement accuracy	0.30	0.39	0.41	0.51
Relative improvement	0.49	0.51	0.44	0.39

## Data Availability

The dataset can be obtained from the corresponding author upon request.
